# Current Knowledge about the Effect of Nutritional Status, Supplemented Nutrition Diet, and Gut Microbiota on Hepatic Ischemia-Reperfusion and Regeneration in Liver Surgery

**DOI:** 10.3390/nu12020284

**Published:** 2020-01-21

**Authors:** María Eugenia Cornide-Petronio, Ana Isabel Álvarez-Mercado, Mónica B. Jiménez-Castro, Carmen Peralta

**Affiliations:** 1 Institut d’Investigacions Biomèdiques August Pi I Sunyer (IDIBAPS), 08036 Barcelona, Spain; cornide@clinic.cat (M.E.C.-P.); monicabjimenez@hotmail.com (M.B.J.-C.); 2 Department of Biochemistry and Molecular Biology II, School of Pharmacy, University of Granada, 18071 Granada, Spain; alvarezmercado@ugr.es; 3 Institute of Nutrition and Food Technology “José Mataix,” Center of Biomedical Research, University of Granada, Avda. del Conocimiento s/n, 18016 Armilla, Granada, Spain; 4 Instituto de Investigación Biosanitaria ibs, GRANADA, Complejo Hospitalario Universitario de Granada, 18014 Granada, Spain; 5 Centro de Investigación Biomédica en Red de Enfermedades Hepáticas y Digestivas (CIBERehd), 08036 Barcelona, Spain

**Keywords:** ischemia-reperfusion injury, nutritional status, supplemented nutrition, gut microbiota, partial hepatectomy, liver transplantation

## Abstract

Ischemia-reperfusion (I/R) injury is an unresolved problem in liver resection and transplantation. The preexisting nutritional status related to the gut microbial profile might contribute to primary non-function after surgery. Clinical studies evaluating artificial nutrition in liver resection are limited. The optimal nutritional regimen to support regeneration has not yet been exactly defined. However, overnutrition and specific diet factors are crucial for the nonalcoholic or nonalcoholic steatohepatitis liver diseases. Gut-derived microbial products and the activation of innate immunity system and inflammatory response, leading to exacerbation of I/R injury or impaired regeneration after resection. This review summarizes the role of starvation, supplemented nutrition diet, nutritional status, and alterations in microbiota on hepatic I/R and regeneration. We discuss the most updated effects of nutritional interventions, their ability to alter microbiota, some of the controversies, and the suitability of these interventions as potential therapeutic strategies in hepatic resection and transplantation, overall highlighting the relevance of considering the extended criteria liver grafts in the translational liver surgery.

## 1. Introduction

An ischemic period is commonly required during hepatectomy or transplantation to avoid possible bleeding or blood transfusions. However, reduction of blood flow damages the liver and impairs liver regeneration [1]. Although ischemia-reperfusion (I/R) injury is commonly associated with poor post-operative results after liver surgery [2], no effective strategies are currently available to resolve this clinical problem. The mechanisms responsible for I/R injury are extremely complex, different depending on the liver type (steatotic versus non-steatotic), and involve a wide range of different cells and pro-inflammatory mediators [1,2,3,4,5,6]. Warm ischemia is associated with hepatic resections, and warm and cold ischemia is associated with liver transplantation (LT). The type of ischemia must be distinguished due to existing debate about the specific pathophysiological mechanisms of each surgical procedure. Other factors to be characterized in I/R injury are the percentage and duration of hepatic ischemia applied and the presence of regeneration (associated with hepatic resections) [7,8]. Steatotic livers have been demonstrated to be less tolerant of I/R injury than non-steatotic livers; therefore, the presence of fatty infiltration in the liver is associated with poor outcome following surgery [9,10,11,12]. Steatotic LT shows increased rates of graft failure compared with the post-operative outcomes of non-steatotic LT [9,13,14]. Similarly, complication rates following resection are two–three-fold higher in patients with hepatic steatosis [10,15]. Given the increasing prevalence of steatosis, and consequently the increase in the number of steatotic livers subjected to surgical conditions [16], the development of protective strategies in liver surgery are required.

Recent advances suggest new concerns about the pathophysiology of hepatic I/R injury. Preexisting nutritional status might affect the post-operative metabolism, liver function, inflammation, and regenerative capacity [17,18]. Starvation exacerbates warm ischemic injury due to the amount of glycogen stored in the liver [19,20,21,22]. Adenosine-5′-triphosphate (ATP) depletion during ischemia induces an acceleration of glycolysis [23]. Although glycolysis is essential for cell survival, its effects may also be detrimental due to lactate accumulation [23]. Overnutrition and specific diet factors are crucial for the pathogenesis and progression of nonalcoholic fatty liver disease (NAFLD) or nonalcoholic steatohepatitis [24]. Although there have been a wide variety of experimental studies on factors and nutritional substrates supporting or inhibiting liver regeneration after resection, a limited number of clinical studies have been addressed [25]. The intestinal microbiota is important to regulate liver functions [26,27] and is crucial in the pathogenesis of NAFLD [28,29,30]. Dietary components, host-intrinsic factors of the gastrointestinal tract affect microbial composition [27,31]. The activation of innate immunity and inflammation caused by gut-derived microbial compounds can exacerbate I/R injury or impair regeneration after liver resections.

The aim of the present review was to summarize the current knowledge from 2014 to 2019 about the effect of starvation, nutritional interventions, and gut microbiota alterations on morbidity and mortality in both experimental and clinical studies of liver surgery. A clear distinction between warm and cold I/R injury (associated with liver resections and LT, respectively) is discussed. The complicated differentiation on experimental models using steatotic and non-steatotic livers is addressed to elucidate the mechanisms responsible of liver I/R injury and for the establishment of new targets and protective strategies. The different results regarding the potential benefits of starvation, nutritional diets, and gut microbiota alterations in different studies (experimental, translational, and clinical studies) in hepatic surgery are discussed. All of this might be useful for the design of appropriate experimental models and treatments in clinical liver surgery.

## 2. Starvation Effects on I/R Injury Associated with Liver Surgery

Experimental studies have shown that liver I/R injury is influenced by different nutrients. For instance, protein restriction improved hepatic I/R injury by up-regulating hydrogen sulfide [32]. The supplementation of vitamins C and E in the diet protected against hepatic I/R injury. This effect was exerted by the up-regulation of antioxidant enzymes as well as the down-regulation of cell adhesion molecules [33]. However, although these experimental studies have demonstrated some beneficial effects of pre-operative diet restriction/fasting in liver I/R injury, the underlying mechanisms remain to be clarified. Other findings are contradictory [34,35,36]. Experimental studies have shown that fasting exacerbates normothermic ischemic injury [19,20,21,22]. Therefore, to support the clinical translation of starvation, the mechanisms behind the fasting-induced protection against I/R injury need to be elucidated [37]. Nil per os (NPO) status in patients undergoing hepatectomy to avoid potential problems, potentially associated with the general anesthesia, may be associated with immunomodulation risks to patients [38,39]. The NPO-associated fasting induces inflammatory responses in surgery [40]. The fasting state results in hyperglycemia, post-surgical infections, and increased length of stay [41,42,43,44]. Similarly, in clinical transplantation, donor starvation because the prolonged hospitalization or lack of an appropriate nutritional support would favor hepatic damage and primary nonfunction [45].

### 2.1. Studies of Short-Term Starvation (12–24 h)

The most recent preclinical studies investigating the effects of short-term starvation (12–24 h) on experimental models of normothermic I/R injury are summarized in Table 1. Twelve hours’ fasting protected against apoptosis and necrosis associated with I/R injury [46]. Higher levels of serum β-hydroxybutyric acid (BHB) and, consequently, forkhead box protein O1 (FOXO1) over-expression were detected following the 12 h fast, thereby increasing antioxidant mechanisms including heme oxygenase 1 (HO-1) and autophagy activity. BHB inhibited the nucleotide oligomerization domain-like receptor family, pyrin domain containing 3 (NLRP3) inflammasome activity, the high-mobility group box 1 (HMGB1) release, and nuclear factor κ-light-chain-enhancer of activated B cells (NF-κB) activation [46]. In an ex vivo perfused rat liver model based on 60 min of ischemia and 60 min of reperfusion, the authors reported that starvation for 18 h fails to provide protection against liver I/R injury. The benefits of feeding were explained, at least partially, by increased energy metabolism (availability of energetic substrates) such as glycogen and high ATP levels [47]. These contradictory results [46,47] could be explained by the use of different experimental models of I/R (in vivo and ex vivo, respectively).

Short-term fasting for 24 h protected against hepatic I/R injury by regulating the response of innate immune cells [37]. Authors have shown that such benefits might be explained by the reduction in the circulating HMGB1 levels, which induces changes in sirtuin 1 (Sirt1) and autophagy, resulting in the anti-inflammatory regulation of short-term fasting [37]. In contrast with the results obtained in the ex vivo perfused rat liver model after 18 h fasting [47], the authors failed to find a correlation between the energy parameters, such as hepatic glycogen stores and fasting-induced protection. Altogether this suggests the relevance of using in vivo I/R models that simulate the clinical conditions as much as possible.

Qin et al. showed that starvation for 24 h inhibited hepatic I/R damage [48]. The authors suggested that starvation had anti-apoptotic effects in I/R by increasing the expression of anti-apoptotic protein such as B-cell lymphoma (BCL)-2/BCL-xl/phospho-protein kinase B (P-Akt) and decreased caspase-3 activity [48]. Similar to Rickenbacher et al. [37], the authors also concluded that starvation induced autophagy in the liver via the Sirt1 pathway [48]. Therefore, the results obtained in preclinical studies of fasting for 24 h suggest that starvation reduces cell death during hepatic I/R. Fasting-activated Sirt1 induced autophagy and promoted anti-apoptosis [48].

In the clinical context, liver resection is usually carried out under vascular occlusion to regulate bleeding [51]. Regeneration affects the mechanisms responsible of I/R injury, and I/R negatively affects liver regeneration. Thus, the beneficial effects of starvation reported to date might not be extrapolated to surgical conditions requiring partial hepatectomy (PH) under I/R.

To the best of our knowledge, only Zhan et al. [49] recently analyzed the effects of short-term fasting on PH under I/R in humans (Table 1). Thus, in a prospective, single-blinded, randomized study of 30 patients per group, 24 h fasting reduced damage, inflammation, and oxidative stress through regulation of nuclear factor erythroid-derived 2-related factor 2 (Nrf2), HO-1, and NAD(P)H quinone dehydrogenase 1 (Nqo1) signaling pathways [49]. However, postsurgical complications of control and fasting groups were similar [49]. Further clinical studies are required to confirm the benefits of 24 h of fasting in PH.

### 2.2. Studies of Long-Term Starvation (Two to Seven Days)

In addition to the investigations on the effects of short-term fasting for 24 h, Rickenbacher et al. [37] and Qin et al. [48] studied the effects of long-term starvation for two and three days (Table 1). Rickenbacher et al. showed that fasting for 24 h, but not two or three days, can reduce I/R injury via the Sirt1-mediated down-regulation of HMGB1 in circulation [37]. However, Qin et al. [48] found even more protective effects against I/R injury at two and three days of fasting than 24 h of fasting in mice. The reasons for these different findings may be related to the different experimental model used, such as duration of ischemia (60 min versus 90 min of ischemia). Three days of fasting or one week of preoperative protein/energy restriction decreased transaminases and hemorrhagic necrosis after 30 min of ischemia [50].

Further experimental investigations and clinical trials are needed to determine the effects of starvation and the exact fasting duration (one, two, or three days) to produce the greatest advantages in patients. Long-term diet restriction (more than 24 h) may be difficult to apply for human preoperative management. Experimental models that reproduce the clinical conditions might be useful for the implementation of protective treatments in clinical conditions in the short-term [52]. The studies mentioned above have been reported in non-steatotic livers. The prevalence of obesity ranges from 24% to 45% of the population; therefore, increases in the number of steatotic livers subjected to liver surgery are expected. Steatotic livers show poor regenerative response and increased vulnerability to I/R injury, and the mechanisms involved in the I/R pathology and protective strategies are different depending on the type of the liver (presence or absence of steatosis) submitted to surgery. Thus, future research in experimental models of PH with I/R and LT are required to understand the underlying mechanisms of starvation, especially in sub-optimal livers in order to ameliorate the viability of livers subjected to surgery and reduce consequently the post-operative problems.

## 3. Nutritional Support by Nutraceuticals and Functional Foods on Liver Surgery under Hepatic Ischemia-Reperfusion

The preoperative nutritional state considerably affects postoperative metabolism, organ function, and inflammatory responses [17], and nutritional status affects the liver regenerative capacity [18]. Therefore, the basal alimentary condition of the patient plays an important role in predicting postoperative complications. Patients with end-stage liver diseases who undergo LT usually present with malnutrition, which directly impacts the deterioration of the patient’s clinical condition, affecting post-transplantation survival [24]. The post-transplantation survival is even more relevant in the case of liver steatosis (the main feature of NAFLD) as these organs show high vulnerability to I/R injury and regenerative failure in comparison with non-steatotic livers [53].

As mentioned above, coinciding with the progressive adoption of the Western lifestyle and changes in nutritional habits, many studies have evidenced the increased incidence and prevalence of NAFLD and other related disorders [54]. Also, malnutrition induces dysbiosis with translocation of bacteria- and/or pathogen-derived components from the gut to the liver [55].

Conversely, several dietary components significantly benefit health [56], presenting antioxidant or anti-inflammatory properties as well as contributing to modifying the gut microbiome [18]. As a result, the re-establishment and maintenance of the correct nutritional status by these nutraceuticals and functional foods before, during, and/or after surgery could lead to improvements in complications related to I/R injury, representing a potential approach alone or in combination with other therapies to improve patient outcomes. Eventually, strategies based on nutrition support could become a major adjunct to the conventional management of I/R injury.

Combination of different nutrition tools like anthropometry, and body composition analysis, have been reported to formulate a composite score for malnutrition assessment [57]. The goals of nutritional therapy are mainly focused on improving protein malnutrition and regulate nutrient deficiencies. Studies to address I/R injury complications by dietary supplementation and functional foods in liver surgery covering 2014 to 2019 are summarized in Table 2.

### 3.1. Plant-Derived Supplements and Other Food Additives

Three studies focusing on nutrition support based on plant-derived supplements and other food additives were reported from 2014 to 2019 [58,59,60]. All of them targeted oxidative stress and inflammatory responses related to I/R injury in murine models. The more remarkable findings were strengths of the antioxidant defense systems and anti-inflammatory properties after the intervention. For instance, ankaflavin, a traditional food additive used in Eastern Asia and China, significantly decreased the proliferation of Kupffer cells and the protein expression of inflammatory cytokines (tumor necrosis factor α (TNF-α), interleukin (IL)-6, and IL-1β) and reduced apoptosis and liver steatosis in high-fat-diet-fed mice [58].

A similar plant-derived strategy tested the potential benefits of apocynin (4-hydroxy-3-methoxyacetophenone) in rats under I/R injury. In this case, a single dose of apocynin 30 min before surgery induced the production of superoxide dismutase (SOD), reduced lipid peroxidation, and decreased glutathione (GSH) limiting the cellular stress triggered by ischemia [59]. Also, Korean red ginseng extract, which contains ginsenosides, phenolic compounds, polysaccharides, and polyacetylenes, showed a chemopreventive effect through antioxidant, apoptotic, and anti-cell proliferation in various cancers. In concordance with these findings, a study conducted in rats in which hepatic cancer had previously been induced, supplementation starting two weeks before surgery and eight weeks after PH revealed chemopreventive effects by prevention of oxidative stress and regulation of redox-enzymes [60]. The potential limitation of all these studies is related to the limited specificity of the different plant-derived supplements and additives. The relevance of the changes on oxidative stress, TNF-α, IL-6, and/or IL-1β induced by such treatment requires further investigation. Studies aimed at evaluating if such benefits can be extrapolated in steatotic liver undergoing surgery might be of clinical and scientific relevance. The potential toxicity and side effects of these components, dependent on the concentrations, required to confer protection should be investigated.

### 3.2. Vitamins

Various vitamins deficiencies have been reported in receptors submitted to LT. Folate deficiency is caused by a decreased intake and absorption, dysregulation in renal excretion and limited hepatic storage. Folate and B12 supplementation is crucial to protect liver against alcoholic hepatitis [75]. Hypovitaminosis A is associated with impairment in immune function and increased risk of fibrosis, which are risk factors in liver surgery [76]. An anti-oxidative nutrient-rich enteral ordinary diet enhanced with vitamins C and E and supplemented with polyphenols (a combination of catechin and proanthocyanidin) for seven days before ischemic insult in mice was able to mitigate liver I/R injury, improving antioxidant and inflammatory parameters that reduced hepatocellular damage [33].

Dexpanthenol, also known as pro-vitamin B5, is oxidized to pantothenic acid (PA), which increases GSH content, coenzyme A (Co A), and ATP synthesis, thus playing a crucial role against oxidative stress and inflammation. In an experimental model of hepatic I/R in rats, a single dose of dexpanthenol before I/R induced the suppression of oxidative stress and increased antioxidant levels [61]. In a swine model of multiple injuries including I/R injury and hemorrhage, the authors observed a moderate improvement in coagulation dysfunction after intravenous provision of high-dose vitamin C and a reduction in proinflammatory/procoagulant response [62].

All these studies indicate the potential importance of vitamins in reducing the inflammation and damage in surgical conditions of I/R. The usefulness of vitamins in the presence of steatosis and in surgical conditions requiring ischemia and regeneration, such as liver resection or liver-related LT, remains to be elucidated.

### 3.3. Fish and Rosa Mosqueta Oils

Based on the well-established protective components of rosa mosqueta oil (i.e., α-linolenic acid (ALA) and tocopherols), Dossi et al. reported that rosa mosqueta oil supplementation before the induction of I/R in rats increased liver ALA and its derived eicosapentaenoic acid (EPA) and docosahexaenoic acid (DHA) fatty acid contents, with increases in α- and γ-tocopherols, normalized liver oxidative stress parameters, and ameliorated liver and serum inflammation indexes [63].

Fish-oil-supplemented diets have been shown to reduce I/R injury. In this sense, a study conducted to identify the effect of tilapia fish oil, which is rich in unsaturated fatty acids, administrated to rats by gavage during three weeks before I/R revealed that after ischemia and 1, 12, and 24 h of reperfusion, antioxidant enzyme activities of catalase (CAT), SOD, and glutathione peroxidase (GPx) decreased in the intervention group. Lipid peroxidation and liver damage decreased in this group [17]. Similarly, daily oral supplementation for 12 days with fish oil, comprising 40% DHA and 40% EPA, induced AMP-activated protein kinase (AMPK) activation and promoted the recovery of liver function during PH [64]. The role of each component included in either rosa-mosqueta- or fish-oil-supplemented diets on the mechanisms responsible for hepatic I/R remains unknown. The main mechanism involved in the effects of such treatments on I/R damage remain to be elucidated. This is a potential problem due to difficulties for the establishment of target signaling pathways in liver surgery. The effect of rosa mosqueta and fish oil supplementation in steatotic liver undergoing PH under vascular occlusion as well as in LT should be investigated.

### 3.4. Fatty Acids, Arginine, and Nucleotides

Polyunsaturated fatty acids (PUFAs) are fatty acids with two or more double bonds in their carbon chain. PUFAs can be further categorized according to the location of the first double bond relative to the terminal methyl group: Omega-3 and omega-6 and are characterized by the presence of a double bond three and six atoms away from the methyl terminus, respectively [77]. Long-chain PUFAs (LC-PUFAs), particularly omega-3 LC-PUFAs EPA and DHA, are associated with beneficial health effects [78].

In experimental and clinical studies performed in animals and humans, fatty acids, arginine, and nucleotides have shown the ability to modulate immune and inflammatory responses [18,69]. These nutrients, among others, have been labeled as pharmaconutrients [18].

Supplementation with amino acids, such as arginine, affects urea genesis, gluconeogenesis, and protein synthesis. Diets enriched with these amino acids increases the hepatic catabolism functions [79]. Enteral immunonutrition with arginine reduces the risk of infections in patients submitted to major operations [80]. The supplementation with L-arginine diet in rats hepatectomized was unable to confirm benefits in liver regeneration [65]. Conversely, a similar study using supplementation of L-glutamine in the diet of rats after PH revealed an increase in the amount of albumin and beneficial effects for liver regeneration [66]. Glutamine favors liver regeneration [66].

Omega-3 fatty acids affect the production of pro-inflammatory mediators, such as growth factors, chemokines, and matrix proteases, showing anti-inflammatory and immunomodulatory effects due to their rapid incorporation into cell membranes [67,68]. However, their effect on regeneration in livers undergoing resection has not been widely reported. Two studies evaluated whether omega-3 fatty acids protect against regeneration failure in PH in rats. Neither long-term supplementation before surgery [67] nor a preoperative supplementation plus the same dose every 24 h during the seven days post-surgery [18] showed any influence on the liver regeneration.

Concerning EPA, a study conducted in patients who underwent major hepatobiliary resection reported that preoperative immunonutrition decreased inflammation and protected against post-surgery infections and complications [68]. However, these benefits cannot be exclusively attributed to EPA because the oral supplementation was also enriched with arginine and nucleotides. A similar approach but with controversial results was conducted by Russell et al. Indeed, any benefit of preoperative immunonutrition was reported with arginine and n-3 fatty acids [69]. In a retrospective study reported by Kamo et al., liver recipients suffering from infection after LT were submitted to enteral immunonutrition enriched with nucleotides, arginine and omega-3 fatty acids, and hydrolyzed whey peptide (HWP) (an immunonutritional liquid). The main finding was a lower incidence of bacteremia in the intervention group compared with the control group [70].

For steatotic livers, Nii et al. tested the effects of HWP on hepatic I/R injury in rats with steatotic livers administered immediately after reperfusion and every six hours thereafter. This treatment ameliorated liver damage, improving function, histology, and survival following I/R [71]. In conditions of PH under I/R, a lipid emulsion comprising 52% linoleic acid, 22% oleic acid, 3% palmitic acid, 8% linolenic acid, 4% stearic acid, 1% other fatty acids, 8.184 g/L egg phospholipids, and 15 g/L glycerine infused in rats immediately after surgery for four hours protected against damage and regenerative failure [72].

### 3.5. Branched-Chain Amino Acid

A branched-chain amino acid (BCAA) is an amino acid with an aliphatic side-chain with a branch. BCAAs promote protein synthesis and glucose metabolism and are involved in fatty acid oxidation [81]. BCAAs favor liver regeneration, nutrition status, and hepatic encephalopathy. BCAAs have the ability to reduce oxidative stress and liver inflammation as well as lactate production [73].

A randomized controlled trial conducted in patients submitted to hepatectomy showed that supplementation with BCAAs administered two times a day for six months after surgery improved liver functionality and regenerative capacity [74]. Similarly, in patients submitted to liver resection, the preoperative BCAA supplementation decreased blood lactate, which is exacerbated by surgical stress patients [73].

### 3.6. Probiotics

Probiotics are cultures of single or multiple microbes that can regulate the properties of the existing gut microbiota. Probiotics can promote anti-inflammatory effects in gut, thereby preventing bacterial translocation and endotoxin generation [82] and are involved in the synthesis of antimicrobial agents that inhibit the invasion of pathogenic bacteria [83]. Probiotics might regulate the immune system, inhibiting the release of cytokines like TNF-α [84] and inducing the release of anti-inflammatory cytokines like IL-10 and tumor growth factor β (TGF-β) [85].

Current evidence has indicated the advantages resulting from the use of probiotics to prevent the infections after LT, as well as to improve the circulatory diseases associated with cirrhosis, hepatic encephalopathy, and Child–Pugh class [86,87]. The improvement in the neutrophil phagocytic capacity induced by probiotics regulated the infections, preventing bacterial translocation. These effects resulted in the restoration of the immune system [88,89,90].

In addition to the different types of nutritional support, the routes of administration should be considered. Oral intake is the first line therapy used to treat malnutrition and decrease the complications (hepatic encephalopathy, infections, and ascites among others) in liver diseases. However, the impact on survival remain to be elucidated [91,92]. It has been described that an increased dietary intake by oral nutrition improved liver function and lowered mortality compared with the enteral and parenteral nutrition [93,94]. Hasse et al. [95] demonstrated early enteral feeding beneficial effects like improved nitrogen balance and fewer viral infections associated with LT. Parenteral nutrition might be used as a second line approach in those who cannot be fed adequately by the oral or enteral route for instance in patients with unprotected airways and advanced hepatic encephalopathy [96,97]. All these data are not conclusive for selecting the most appropriate administration route of nutritional support. In a comparison between parenteral and early enteral nutrition, both strategies were equally effective to the maintenance of nutritional state [97]. The European Society for Parenteral and Enteral Nutrition (ESPEN) guidelines for organ transplantation recommend enteral nutrition or oral nutritional supplementation to improve nutritional status and liver function [93,98,99,100,101]. Enteral nutrition reduces the incidence of viral and bacterial infections. For enteral nutrition, the ESPEN guidelines recommend the use of more concentrated high-energy formulas in patients with ascites and BCAA-enriched formulas in hepatic encephalopathy patients [95].

## 4. Gut Microbiota and Hepatic Ischemia Reperfusion in Liver Surgery

The gut microbiota is crucial to the effects of diet, drugs, and disease [102]. The microorganisms that exist within the gastrointestinal ecosystem are termed gut microbiota, playing an essential role in the stimulation of immune response [103], the maintenance of intestinal barrier integrity [104], modulation of host–cell proliferation and vascularization [105,106], and regulation of neurological [107] and endocrine [108] functions. The human gut microbiota provides an energy source [109], is involved in the synthesis of vitamins and neurotransmitters [110], metabolizes bile salts [111], and eliminates toxins [112].

**Figure 1:**
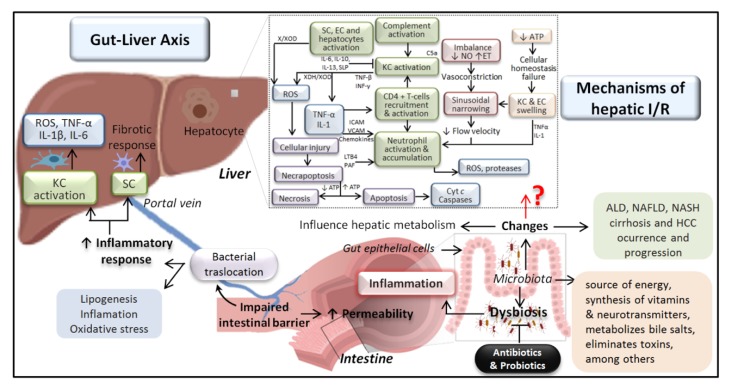
Gut microbiota and hepatic I/R. The dotted box summarizes the mechanisms involved in hepatic I/R injury and how some of these have been altered in the liver by changes in the gut microbiota. ALD, alcoholic liver disease; ATP, adenosine triphosphate; Cyt c, cytochrome c; EC, endothelial cell; ET, endothelin; HCC, hepatocellular carcinoma; ICAM, intracellular cell adhesion molecule; IL, interleukin; INF, interferon; KC, Kupffer cell; LTB4, leucotriene B4; NAFLD, nonalcoholic fatty liver disease; NASH, nonalcoholic steatohepatitis; NO, nitric oxide; PAF, platelet activating factor; ROS, reactive oxygen species; SC, stellate cell; TNF, tumor necrosis factor; VCAM, vascular cell adhesion molecule; and X/XOD, xanthine/xanthine oxidase.

Disequilibrium in the microbiota composition, commonly referred to as dysbiosis, may lead to several diseases [113,114]. The gut and liver (the gut–liver axis) (Figure 1) communicate bidirectionally through the biliary tract, the portal vein, and the systemic circulation [115]. The translocation of bacterial products from the intestine to the liver induces inflammation in different cell types, such as Kupffer cells and a fibrotic response in hepatic stellate cells, resulting in deleterious effects on hepatocytes [116]. Bacterial translocation and fungal cell wall components are increased in experimental models of ethanol-induced liver disease [117].

Alterations in gut microbiota are important for determining the occurrence and progression of alcoholic liver disease (ALD) [118,119,120], NAFLD [121,122], nonalcoholic steatohepatitis (NASH) [123,124], cirrhosis [125,126], and hepatocellular carcinoma (HCC) [127]. Fecal microbiota transplantation could induce hepatitis B virus e-antigen (HBeAg) clearance in patients with persistent positive HBeAg, even after long-term antiviral treatment [128]. Ferrere et al. [129] observed that ALD in mice were reduced by fecal transplantation from alcohol-fed mice resistant to ALD or with prebiotics.

Evidence points to the involvement of the gut microbiota in the pathogenesis of NAFLD [130,131]. Cogger et al. showed that liver sinusoidal endothelial cells (LSECs) fenestrae are inversely and positively correlated with the gut abundance of Bacteroidetes and Firmicutes, respectively [132]. The gut microbiota also has an emerging role in NASH as a source of inflammatory stimuli [130,133]. Increased intestinal permeability and elevated plasma lipopolysaccharide (LPS) [134,135] observed in NASH may also contribute to LSECs’ pro-inflammatory function [136].

Gut microbiota shifts the influence of hepatic metabolism through regulation of hepatic gene expression without direct contact with the liver [137,138].

As a result, ischemia produced during liver surgery (i.e., LT or liver resection) is expected to alter the microbiota profile, potentially affecting inflammation, the immune response, and even regeneration. The gut–liver axis is widely implicated in the pathogenesis of liver diseases such as NAFLD, NASH, HCC, and acute liver failure [139]. The gut microbiota may also contribute to the generation of memory alloreactive T cells. T cells were reported to be important in transplant rejection and many experimental and clinical studies have shown that the intestinal microbiota is altered after allogeneic transplantation [140].

In the context of I/R injury, hepatic steatosis is a key factor to consider due to negative influences on patients’ outcomes [141]. Gut microbiota fundamentally influences processes such as lipogenesis, which is affected by the absorption of monosaccharides in the intestinal lumen by the microbiota [142], and bile acids, since they are able to de-conjugate them and turning them into secondary bile acids, which are capable of interacting with a nuclear receptor of the farnesoid receptor X [143]. Changes in gut microbiota promote the development of NAFLD since affect inflammation, insulin resistance, bile acids, and choline metabolism. The Western diet is associated with intestinal microbial dysbiosis [144] and the development and prevalence of NAFLD [145]. I/R injury is a common cause of rejection when grafts are sourced from NAFLD donors; the prevalence of the problem is increasing [141].

The gut microbiota alterations in NAFLD patients remain to be characterized [114]. Several reviews have highlighted studies focused on strategies to prevent and target gut microbiota (probiotics, prebiotics, diet or fecal microbiota transplantation, among others) in NAFLD [114,115,140,146]. Others have addressed the management of nutrition in patients with end-stage liver disease undergoing LT [146,147]. However, studies evaluating changes in gut microbial populations and diversity caused by hepatic I/R and their consequences in liver function and regeneration are limited. From 2014 to 2019, authors only examined the effect of therapeutic approaches on intestinal microbiota and hepatic injury and such strategies were mainly based in the use of antibiotics. Despite this, the effects of antibiotics on hepatic damage being caused by regulation of the intestinal microbiota remain to be clarified. None of these studies aimed to improve damage induced by I/R in steatotic livers.

Intestinal microbial characterization and alteration in early phase and subsequent intestinal barrier dysfunction during acute rejection after LT have been reported [148,149,150,151,152,153]. Due to the high sensitivity of microbial changes during acute rejection after LT, intestinal microbial variation has been suggested to predict acute rejection in the early phase after LT [148]. Therefore, gut microbial profiles have been suggested as predictive injury biomarkers in LT [153].

Gut microbiota might affect immune mediators such as IL-6 and regulate liver regeneration. Following the administration of antibiotics (Table 3), the number of CD1d-dependent natural killer T (NKT) cells was reduced after partial hepatectomy (PH) [154]. NKT cells and activated Kupffer cells produced high levels of interferon-γ (IFNγ) and IL-12. Thus, antibiotic administration after PH could negatively affect regenerative response [154]. It has been reported that PH resulted in an upregulation of more than 6000 bacterial genes, some of them involved in regeneration and was also accompanied by changes in the gut microbiota (e.g., an increase in *Bacteroidetes* and *Rikenellaceae,* and decreases in *Clostridiales*, *Lachnospiraceae*, and *Ruminococcaceae*) [155,156].

The administration of antibiotics reduces hepatic injury in rats submitted to LT with acute rejection, but the microvilli of the ileum epithelial cells were destroyed, inducing alterations in microbiota [157]. Further studies are required for a more understanding of the immunity interactions between gut microbiota and the rejection after LT [157]. Two retrospective studies support the notion that antibiotics (rifaximin, neomycin, erythromycin, and ampicillin-sulbactam) administration prior to LT reduce infections associated with LT, thus reducing the liver injury, inflammation, and early allograft dysfunction [158,159]. However, further randomized controlled clinical trials are required to elucidate the exact mechanisms of action of such antibiotics, their target signaling pathways, and the optimal duration of treatment. Further experiments in animal LT models will be required to elucidate the specific molecular signaling pathways through which antibiotics may exert their actions, as well as to investigate whether the protection on hepatic damage induced by the treatment with antibiotics is exerted throughout changes in the gut microbiome.

Survival outcomes after LT have constantly improved using upgraded immunosuppressive agents [165]. However, the inadequate or excessive immunosuppression is associated with a higher risk of rejection, higher incidence of infection, drug toxicity, and increased mortality [166,167,168,169,170]. Experimental studies in rats have investigated the effect of immunosuppressive agents on the intestinal microbiota in LT. The results showed that cyclosporine A ameliorated hepatic injury and partially restore the intestinal microbiota after LT [160]. An optimal dosage of tacrolimus (FK506) induced normal graft function, and stable gut microbiota after LT in rats. This resulted in increased probiotics, including *Faecalibacterium prausnitzii* and *Bifidobacterium* spp. and decreased pathogenic endotoxin-producing bacteria, such as the *Bacteroides*–*Prevotella* group and *Enterobacteriaceae*. Thus, the use of the gut microbiota might be a novel strategy for the assessment of the dosage of immunosuppressive medications and its effects in receptors submitted to LT [161].

Retinoic acid, naturally present in the gastrointestinal tract, has a relevant effect in regulating lipid homeostasis [171,172] and can facilitate PH-induced liver regeneration [173,174]. Given the intimate relationship between gut-derived signaling and liver regeneration, authors hypothesized that retinoic acid may regulate gut microbiota thereby promoting liver regeneration [162]. Retinoic-acid-accelerated liver regeneration was associated with a reduction in the ratio of Firmicutes to Bacteroidetes. Retinoic acid had benefits on lipid circulation and regulated the FGF21-LKB1-AMPK pathway, which promoted energy metabolism and consequently the regenerative process in the liver [162]. Further studies will be required to elucidate the interaction between the modulation of microbiota and the improvement in proliferation induced by the retinoic acid. This will allow the development of clinical therapeutic strategies to promote liver regeneration.

In line with the results described above, the evidence suggests that probiotics play an important role in the stability of the intestinal microbiological environment and regulate intestinal microbiota. A double-center and double-blind randomized clinical trial conducted in colorectal liver metastases patients showed that the incidence of infectious complications after preoperative and postoperative supplementation with probiotics decreased blood *Escherichia coli*, *Staphylococcus aureus*m, and *Aeruginosin* populations, improved intestinal barrier function, and reduced postoperative infection rate [163].

As time-restricted feeding (TRF) is a promising intervention against the worldwide trend of obesity and other metabolic diseases [175], a study conducted in mice investigated whether alteration in gut microbiota caused by TRF could alleviate hepatic I/R injury [164]. The results confirmed the adverse effect of I/R on the gut microbial population. However, TRF prior to surgery reduced the damage, oxidative stress, and inflammatory biomarkers associated with I/R, likely due to intestinal increases in Firmicutes phylum, Clostridia and Bacilli classes, Clostridiales and Lactobacillales orders, and Lachnospiraceae and Ruminococcaceae families, which could be hallmarks of a healthy gut [164].

## 5. Future Perspectives and Conclusions

The temporary occlusion of hepatic inflow is commonly used during liver resection or LT, creating an unsolved problem in clinical practice associated with post-operative morbidity and mortality. Experimental studies have shown that liver I/R injury is influenced by various nutrients, suggesting the importance of dietary control for preventing I/R injury.

Today, starvation is not a feasible strategy in clinical practice. Future clinical and preclinical studies on PH with I/R and LT are required to understand the underlying mechanisms of starvation to increase the quality of livers subjected to surgery and reduce the post-operative disorders. Controversial results have been reported in experimental models of starvation under I/R conditions [37,48], which might be explained by the use of different times of ischemia (60 or 90 min). The literature draws upon research data that support the duration of ischemia differentially affects hepatic I/R injury [176,177,178]. This is of clinical interest since, in clinical practice, the timing of ischemia dependent on the complications associated with surgery cannot be predicted, whereas the effects resulting from starvation are dependent on the duration of ischemia and the duration of starvation. In clinical practice, long-term diet restriction of more than 24 h is difficult to apply for preoperative management in LT. Liver donors are often kept in the intensive care unit for periods no longer than six hours after diagnosis of brain death. The time frame between the declaration of brain death and organ procurement provides a shorter window for the starvation intervention. The effects of starvation on steatotic livers undergoing surgery should be evaluated since the mechanisms responsible for I/R and consequently the useful therapeutic strategies in clinical practice might be different in steatotic and non-steatotic livers submitted to surgery. The number of steatotic livers submitted to surgery is expected to increase, though steatotic livers show regenerative failure responses and reduced tolerance to I/R injury compared with non-steatotic livers. Therefore, research in experimental models of PH with I/R and LT that closely reproduce the clinical conditions is required to understand the underlying mechanisms of starvation, especially in sub-optimal livers.

To summarize, several nutrients and dietary supplements have antioxidant or anti-inflammatory properties and contribute to modifying the gut microbiome. These properties might warrant investigations using them as potential strategies to counteract I/R injury complications and promote regeneration from a nutritional point of view. The diagnosis of nutritional status and its re-establishment and maintenance, as well as providing adequate nutritional support during all phases of the surgery, could be considered the first step to formulating adequate I/R injury therapy. From our view, studies using this approach are insufficient, with only 20 studies from 2014 to 2019, with considerable variability in models, time, and administration. This suggests that the effects of such approaches on hepatic I/R injury are specific for each surgical procedure (for instance, warm ischemia associated with hepatic resections versus LT, times of ischemia, and type of treatment: Short or prolonged fasting).

Most studies based on nutrients and dietary supplements reported benefits on liver function and oxidative stress parameters, but we did not find many studies aimed to improve liver regeneration (six of 20) and only three reported improvements in this parameter. As steatotic grafts show increased vulnerability to I/R when they are transplanted and pre-existing steatosis is related with impairment of liver regeneration following PH [53,141], more than the only three studies performed in steatotic liver seems to be warranted. We only found one study reporting the use of probiotics as a strategy. As a dysbiotic microbiota induces the translocation of several bacterial components into the portal vein and favors the activation of innate immunity and inflammation [114], modulation of gut microbiota from a nutritional point of view is mandatory for evaluating and modifying alterations associated with I/R injury and, in consequence, further studies in this area are needed.

In our view, a strategy more appropriate for clinical practice is the re-establishment and maintenance of the correct nutrient deficiencies using nutraceuticals and functional foods before, during, and/or after surgery, dependent on the patient’s requirements. In hepatic resections, this strategy is suitable for the treatment of patients before during or after surgery, whereas in the case of LT, this strategy was only possible after LT with considerable difficulties during liver surgery.

For us, the use of plant-derived supplements, fish, and rosa mosqueta oils show limitations and are inadvisable due their limited specificity and the potential toxicity and side effects of these components. Vitamins, branched-chain amino acid, fatty acids, arginine, and nucleotides can be administered in clinical practice only if deficiencies exist in the patients. Thus, exhaustive studies in patients are required since, for instance, hypervitaminosis is associated with toxic effects. Given the limited studies on the effect of administering vitamins in surgery, conclusions about their efficacy cannot be drawn. Before the administration of fatty acid, the deficiencies in specific types of fatty acid in the patient must be determined. In some cases, for instance EPA supplementation, benefits have been reported but whether the potential benefits are exclusively attributed to EPA is unknown because oral supplementation was also enriched with arginine and nucleotides. Only through exhaustive studies of the patient’s deficiencies can we select the most effective treatment for the patient. Unfortunately, these studies are not performed routinely in clinical practice since, in many cases, surgery is performed an emergency situation but the techniques that evaluate such components are complex, time consuming, and expensive.

Although I/R is known to have detrimental effects on the gut microbial population, studies reporting interventions targeting gut microbiota in the I/R setting are limited. A more accurate characterization of the gut microbiome and host responses using different liver surgery models, stages of liver disease, and larger cohorts of patients is required. A comprehensive understanding of the intestine microbiota’s role during hepatic surgery is lacking. Maintaining the stability and/or restauration of the intestinal microbiological environment could be a safe and sustainable tool for mitigating I/R injury, which could even effect regeneration. Although regulation of the gut microbiota has been primarily achieved through the use of probiotics, as well as through dietary intervention, studies recently reported using mainly antibiotics and mostly focused on avoiding graft rejection and infectious complications post-surgery [148,158,159,163]. Further investigations are required to elucidate whether personalized and precision medicine approaches based on gut microbiota are necessary dependent on the type of surgical procedure. Dose, frequency, and route of modulation of gut microbiota should be addressed.

Probiotics supplementation requires special consideration. This is associated with the regulation of infections by altering gut microbiota and improvements in inflammation and immunological problems associated with liver surgery. Of clinical interest, gut microbial profiles have been suggested as predictive injury biomarkers in LT. However, before the application of probiotics, an exhaustive examination of the alterations in the intestinal microbiota must be performed for the administration of specific probiotics that counteract such deficiencies in the patients. An alternative to the use of probiotics would be the administration of antibiotics. However, the specificity and the appropriate dose must be determined to prevent harmful effects to ileum epithelial cells and the mucosal barrier. Rapid techniques that routinely evaluate intestinal microflora would be necessary if the aim is to establish probiotics as a useful strategy in clinical of liver surgery, especially in LT. Consequently, nutritional support must be personalized based on the patient’s deficiencies. To date, I/R injury is a common complication for patients undergoing liver surgery and its relationship with changes in the gut microbiota is not totally understood. The understanding of such changes and mechanisms involved could help with restoring unhealthy microbial diversity and the richness of species, providing a potential therapeutic tool for treating I/R damage.

## Figures and Tables

**Table 1:** Starvation approach in the setting of ischemia-reperfusion (I/R) injury in studies from 2014 to 2019.

Starvation Time	Model	Specie	Main Therapeutic Effects
Short-term: 12 h	Ischemia WIT: 60 min RT: 0, 1, 3, 6, 12 h [46]	Mice	↓ Liver injury, inflammation, apoptosis ↑ BHB, FOXO1 and HO-1
Short-term: 18 h	Ex vivo Ischemia WIT: 60 min RT: 60 min [47]	Rats	↑ Liver injury, inflammation, apoptosis ↓ Energetic substrates (ATP, glycogen)
Short-term: 24 h	Ischemia WIT: 60 min RT: 6 h [37]	Mice	↓ Liver injury, inflammation, HMGB1 ↑ Sirt1 activity, autophagy
	Ischemia WIT: 90 min RT: 6 h [48]	Mice	↓ Liver injury, inflammation, caspase-3 ↑ Sirt1 activity, autophagy, anti-apoptotic proteins
	Ischemia WIT: 60 min RT: 6 h [49]	Humans	↓ Liver injury, inflammation, oxidative stress ↑ Nrf2, HO-1 and Nqo1
Long-term: 2–3 days	Ischemia WIT: 60 min RT: 6 h [37]	Mice	↑ Liver injury, inflammation, HMGB1
	Ischemia WIT: 90 min RT: 6 h [48]	Mice	↓ Liver injury, inflammation, caspase-3 ↑ Sirt1 activity, autophagy, anti-apoptotic proteins
Long-term: 3–7 days	Ischemia WIT: 30 min RT: 24 h [50]	Mice	↓ Liver injury

Note: ATP, adenosine triphosphate; BHB, β-hydroxybutyric acid; FOXO1, forkhead box protein O1; h, hour; HMGB1, high-mobility group box 1; HO-1, heme oxigenase 1; min, minute; NF-κB, nuclear factor kappa-light-chain-enhancer of activated B cells; Nqo1, NAD(P)H quinone dehydrogenase 1; Nrf2, nuclear factor erythroid-derived 2-related factor 2; RT, reperfusion time; Sirt1, sirtuin 1; and WIT, warm ischemia time.

**Table 2:** Studies to address hepatic I/R injury by dietary supplementation and functional foods.

Drug	Administration	Model	Specie	Main Therapeutic Effects
Ankaflavin (food additive) [58]	Gavage (orally) 0.624 mg/kg daily for 1 week	Ischemia, fatty liver WIT: 60 min RT: 3 h	Mice	↓ Liver injury, steatosis, oxidative stress, apoptosis, inflammatory cytokines (TNF-α, IL-6, IL-1β)
Apocynin (organic compound related to vanillin) [59]	Intraperitoneally 20 mg/kg 30 min before surgery	Ischemia WIT: 60 min RT: 60 min	Rats	↓ Oxidative stress (MPO) ↑ Antioxidant levels (SOD)
Korean red ginseng extract [60]	Orally 0.5%, 1%, or 2% for 10 weeks	PH RT: 7 weeks	Rats	↓ Lipid peroxidation, cytochrome P450 signaling pathway ↑Antioxidant levels (tGSH, GST, GPx),
Antioxidative nutrient-rich enteral diet (Polyphenols, Vitamin C and E) [33]	Orally ad libitum for 7 days	Ischemia WIT: 60 min RT: 6 h	Mice	↓ Liver injury, necrosis, inflammatory cytokines (IL-6, CXCL1), MDA, cell adhesion molecules, neutrophils and macrophage infiltration ↑ Antioxidant levels (SOD1, SOD2)
Dexpanthenol (analogue of provitamin B5) [61]	Intraperitoneally 500 mg/kg during the ischemic period	Ischemia WIT: 60 min RT: 60 min	Rats	↓ Oxidative stress (MPO), histologic tissue damage ↑ Antioxidant levels (SOD, tGSH)
Vitamin C [62]	Intravenous 50–200 mg/kg after surgery	Ischemia WIT: 3 × 15 min pringle maneuver with 5 min between occlusion RT: 4 h	Swine	↓ Inflammatory cytokines (IL-1β, IL-8, TNF-α), procoagulant response (PAI-1, tissue factor)
Rosa mosqueta oil [63]	Orally 0.4 mL/g/day for 21 days	Ischemia WIT: 60 min RT: 20 h	Rats	↓ Liver injury, inflammation, oxidative stress ↑ α-linolenic acid, EPA and DHA fatty acids levels
Tilapia fish oil [17]	Gavage (orally) 0.4% body weight for 3 weeks	Ischemia WIT: 30 min RT: 1, 12, and 24 h	Rats	↓ Liver injury, antioxidant levels (CAT, SOD, GPx), tissue TBARS, histological tissue damage
Fish oil [64]	Gavage (orally) 12 mL/kg daily	PH RT: 1, 2, 3, and 5 days	Mice	↓ Liver injury, total bilirubin ↑ Proliferation, AMPK activation, liver-to-body weight ratio, tight junction, and BSEP protein expression
L-arginine [65]	Gavage (orally) 10% in 1 mL/100g of solution 15 min before surgery and 24 h until date of death	PH RT: 24 h, 72 h, and 7 days	Rats	↑ Alkaline phosphatase No effect in regeneration
L-glutamine [66]	Gavage (orally) 1 mL/100g body weight 6 h and 15 min before surgery	PH RT: 24 h, 72 h, and 7 days	Rats	↑ Regeneration, albumin No effect in liver function
Omega-3 fatty acids [67]	Orally 10 mg/kg/day for 28 days	PH RT: 7 days	Rats	↓ Inflammatory cellular infiltrate No effect in regeneration
Omega-3 fatty acids [18]	Gavage (orally) 1 mL/100g (10% v/v) 15 min and 24 h before surgery	PH RT: 24 h, 72 h, and 7 days	Rats	↓ GGT No effect in regeneration
Immunonutrients (EPA, arginine, and nucleotides) [68]	Orally 1000 kcal/day for 5 days before surgery	PH RT: 1, 3, 7, and 14 days	Humans	↓ Inflammatory response (IL-6), infection, severe complications ↑ Resolving E1
Immunonutrientes (EPA, arginine, and nucleotides) [69]	Orally 3 × 237 mL 1020 kcal, 54 g protein, 12.6 g arginine, 1.3 g nucleotides, 3.3 g EPA/day × 5 days before surgery	PH RT: 1, 3, 5, 7, 10, and 30 days	Humans	No benefits
Immunomodulating diet enriched with HWP [70]	Intravenous 20 mL/h 24 h after surgery	LDLT CIT: 132 ± 100 min RT: 0, 1, 2, 3, and 4 weeks	Humans	↓ Incidence of bacteremia
Hydrolyzed whey peptide (HWP) [71]	Orally 4 mL every 6 h after reperfusion	Ischemia, steatotic liver WIT: 30 min RT: 6 and 12 h	Rats	↓ Liver injury, inflammatory cytokines (TNF-α, IL-6), iNOS, oxidative stress (UCP-2), necrosis ↑ Survival
Lipid emulsion [72]	Intravenous 5 mL 4 h after surgery	PH + I/R, steatotic liver WIT: 60 min RT: 12, 24, and 48 h	Rats	↓ Liver injury, TGF-β ↑ Regeneration (HGF, cyclin A and E), IL-6, ATP, phospholipid levels
BCAA [73]	Orally 1000 mg valine, 2000 mg leucine, 1000 mg isoleucine in 500 mL until 2 h before surgery	PH RT: 0 day	Humans	↓ Lactate levels No effect in morbidity rates
BCAA [74]	Orally 4 g BCAA granules with: 952 mg L-isoleuciene, 1904 mg L-leucine, 1144 mg L-valine twice daily for 6 months	PH RT: 1–2 weeks until 1, 3, and 6 months	Humans	↑ Functional regeneration No effect in infectious, nutritional and immunologic status

Note: AMPK, AMP-activated protein kinase; ATP, adenosine triphosphate; BCAA, branched chain amino acids; BSEP, bile salt export pump; CAT, catalase; CXCL1, chemokine ligand 1; DHA, docosahexaenoic acid; EPA, eicosapentaenoic acid; GGT, gamma glutamyltransferase; GPx, glutathione peroxidase; GST, glutathione s-transferases; HGF, hepatic growth factor; HWP, hydrolyzed whey peptide; I/R, ischemia reperfusion; IL, interleukin; iNOS, nitric oxide synthase; LDLT, living donor liver transplantation; mg, milligram; min, minutes; MPO, myeloperoxidase; PH, partial hepatectomy; PAI-1, plasminogen activation inhibitor-1; RT, reperfusion time; S1P, sphingosine-1-phosphate; SOD, superoxide dismutase, TBARS, thiobarbituric acid reactive substances; TGF-β, tumor growth factor β; tGSH, total glutathione; TNF-α, tumor necrosis factor α; UCP2, uncoupling protein 2; and WIT, warm ischemia time.

**Table 3:** Therapeutic strategies in modulation of gut microbiota in liver surgery from 2014 to 2019.

Drug	Administration	Model	Specie	Main Therapeutic Effects
Ampicillin, neomycin sulfate, metronidazole and vancomycin [154]	Orally 1 g/L ampicillin, neomycin sulfate, metronidazole, and 500 mg/L vancomycin for 4 weeks	PH	Mice	↓ Liver regeneration ↑ IFNγ, IL-12
Gentamicin [157]	Gavage 2 mL daily for 3 weeks	LT CIT: Not indicated RT: 1 week and 2 weeks	Rats	↓ Liver injury, necrosis, inflammation
Rifaximin [158]	Orally 550 mg twice daily for 28 days	LT CIT: 440 min RT: not indicated	Humans	↓ Liver injury, inflammation, early allograft dysfunction
Amoxicillin [159]	Gavage 50 mg/mL for 10 days before LT	LT CIT: 18 h RT: 6 h	Mice	↓ Liver injury, inflammation, CHOP, mTORC1 activity ↑ PGE2, EP4, autophagy
Neomycin, erythromycin and ampicillin-sulbactam [159]	Orally 1 g neomycin, erythromycin 4× and 3 g ampicillin-sulbactam before or on day of LT	LT CIT: not indicated RT: not indicated	Humans	↓ Liver injury, inflammation, CHOP, early allograft dysfunction ↑ EP4, LC3B, autophagy
Cyclosporine A [160]	Intragastrically 2 mg/kg twice daily for 28 days after LT	LT CIT: not indicated RT: 28 days	Rats	↓ Liver injury, inflammation
Tacrolimus [161]	Subcutaneously, 1.0, 0.5, or 0.1 mg/kg every 12 h for 7 days and intragastrically once daily for 8–29 days after LT	LT CIT: not indicated RT: 30 days	Rats	↓ Liver injury
Retinoic acid [162]	Gavage 25 μg/g body weight 48 h before surgery	PH	Mice	↑ Liver regeneration, FGF21
Probiotics [163]	Orally 2 g/day LP, LA-11, and BL-88, total of 2.6 × 10^14^ CFU daily for 6 days before surgery and 10 days after surgery	PH RT: 10 days	Humans	↓ Infectious complications, septicemia, plasma endotoxin, serum zonulin concentration ↑ Liver barrier
Time-restricted feeding [164]	Food restriction: 8–10 h/day, 12 weeks before surgery	Ischemia WIT: 60 min RT: 6, 12, 24 h	Mice	↓ Liver injury, inflammation, oxidative stress, apoptosis

Note: BL-88, *Bifido-bacterium longum* 88; CFU, colony forming units; CHOP, CCAAT/enhancer-binding protein homologous protein; CIT, cold ischemia time; EP, prostaglandin E2 receptor; FGF21, fibroblast growth factor 21; IFNγ, interferon-gamma, IL, interleukin; LA-11, *Lactobaciullus acidophilus* 11; LC3B, Light Chain 3 isoform B; LP, *Lactobacillus plantarum*; LT, liver transplantation; mTORC1, mammalian target of rapamycin complex 1; PGE2, prostaglandin E2; PH, partial hepatectomy; RT, reperfusion time; and WIT, warm ischemia time.

## Abbreviations

AKT	Protein kinase B
ALA	α-linolenic acid
ALD	Alcoholic liver disease
AMPK	AMP-activated protein kinase
ATP	Adenosine triphosphate
BCAA	Branched-chain amino acid
BCL	B-cell lymphoma
BHB	β-hydroxybutyric acid
CAT	Catalase
Co A	Coenzyme A
DHA	Docosahexaenoic acid
EPA	Eicosapentaenoic acid
ESPEN	European Society for Parenteral and Enteral Nutrition
FOXO1	Forkhead box protein O1
GSH	Glutathione
HBeAg	Hepatitis B virus e-antigen
HCC	Hepatocellular carcinoma
HMGB1	High mobility group box 1
HO-1	Heme oxygenase 1
HWP	Hydrolyzed whey peptide
I/R	Ischemia-reperfusion
IFNγ	Interferon-gamma
IL	Interleukin
LC-PUFAs	Long-chain PUFAs
LSEC	Liver sinusoidal endothelial cells
LT	Liver transplantation
NAFLD	Nonalcoholic fatty liver disease
NASH	Nonalcoholic steatohepatitis
NF-κB	Nuclear factor kappa-light-chain-enhancer of activated B cells
NKT	Natural killer T
NLRP3	Nucleotide oligomerization domain-like receptor family, pyrin domain containing protein 3
NPO	*Nil per os*
Nqo1	NAD(P)H quinone dehydrogenase 1
Nrf2	Nuclear factor erythroid-derived 2-related factor 2
PA	Pantothenic acid
PH	Partial hepatectomy
PUFAs	Polyunsaturated fatty acids
Sirt1	Sirtuin 1
SOD	Superoxide dismutase
TGF-β	Tumor growth factor beta
TNF-α	Tumor necrosis factor alpha
TRF	Time restricted feeding
